# Healthcare utilization associated with antimicrobial resistance at a tertiary hospital in Vietnam: A retrospective observational study from 2016 to 2021

**DOI:** 10.1371/journal.pone.0329539

**Published:** 2025-08-04

**Authors:** Nga Thi-Quynh Nguyen, Nhien Phan-Thuy Nguyen, Quynh Thuy Truong, Thao Phuong Huynh, Hong-Nguyen Tran-Thi, Minh Van Hoang, Hai-Yen Nguyen-Thi

**Affiliations:** 1 Department of Pharmaceutical Administration, University of Medicine and Pharmacy at Ho Chi Minh City, Ho Chi Minh City, Vietnam; 2 Department of Pharmacy, Hospital for Tropical Diseases, Ho Chi Minh City, Vietnam; 3 Hanoi University of Public Health, Hanoi, Vietnam; Hawassa University College of Medicine and Health Sciences, ETHIOPIA

## Abstract

**Background:**

Despite the increasing burden of antimicrobial resistance (AMR), specifically on priority ESKAPE pathogens, studies examining the economic impact of AMR in low- and lower-middle-income countries have been scarce and require further investigation to optimize the post-COVID resource allocation.

**Objectives:**

To quantify the incremental hospital costs and length of stay (LOS) associated with antimicrobial-resistant versus -susceptible among priority ESKAPE pathogens from the healthcare sector perspective.

**Methods:**

We conducted a retrospective observational study of patients hospitalized at the Hospital for Tropical Diseases from 2016−2021 with non-duplicate isolates of any ESKAPE pathogens from clinical specimens. The patients were then stratified into resistant- and susceptible- groups by the WHO classification. Multivariate generalized linear regression and negative binomial regression with linear spline at COVID-19 occurrence were employed to evaluate the incremental hospital costs and LOS due to AMR, respectively. These regressions were adjusted for sociodemographic and clinical characteristics. We applied difference-in-difference (DiD) to estimate the differential cost between resistant and susceptible groups regarding COVID-19 change.

**Results:**

During the six-year period, 4,197 out of 6,670 patients (62.92%) were isolated with priority pathogens, with the highest prevalence of priority pathogens observed in 3GCREC and MRSA (accounting for 45.63% and 25.33%, respectively). After covariate adjustments, the incremental hospital costs per resistant patient were significantly higher across most pathogens except for patients tested with MRSA results (average CRAB $3,980; CRPA $1,000; 3GCREC $444; 3GCRKP $1,942; MRSA -$326), while incremental LOS ranged from 1.40 days for 3GCREC (95%CI: 0.69–2.10 ) to 12.54 days for CRPA (95%CI: 11.12–13.97). COVID-19 significantly enlarged the hospital cost gaps between patients with antibiotic-resistant and antibiotic-susceptible profiles, with *A.baumannii* (CRAB vs. CSAB) showing the highest DiD at $9,116 (95%CI: $6,019-$12,213).

**Conclusion:**

The incremental hospital costs of AMR were significant, with the highest one observed in CRAB patients, and the difference between resistant and susceptible cases widened during the COVID-19 pandemic.

## Introduction

Antimicrobial resistance (AMR), which is among the top 10 serious global health concerns of the twenty-first century, has integrated the multifaceted consequences of healthcare with an estimated 192 million DALYs and 4.95 million deaths associated with resistant infections in 2019 [1,2]. The misuse and overuse of antibiotics are key factors in the development of AMR, leading to a problematic situation for the patients due to limited treatment choices, additional diagnostic tests, and prolonged length of stay (LOS). Meanwhile, the health systems also bear the economic burden of heightened infection controls, overconsumption of resource use, and hospital capacity constraints [3]. The review by the British Government [4,5] estimated that $100 trillion in healthcare spending, equivalent to 3.8% of the global gross domestic product (GDP), will be attributed to AMR by 2050 unless we strengthen keypolicy plans and initiatives. Therefore, estimating the accurate costs associated with AMR is essential in providing necessary insights, such as setting a reference point to propose affordable antimicrobial stewardship and infection control approaches, and reducing hospital expenses [6].

Although the emergence of AMR has been disproportionately distributed in low- and middle-income countries (LMIC), studies on the economic impact of AMR in these countries have been scarce due to methodological inconsistencies and data retrieval problems. This inability to quantify the actual economic burden of AMR might hinder the decision-making process for allocating resources effectively [7]. Based on the systematic review by Poudel et al. in 2023 [7], demonstrating the actual cost of resistance is challenging due to a wide range of cost estimates and various methodological assumptions among studies measuring the cost of AMR. Regarding data collection, most data analyzed in published studies were extracted from the synchronous healthcare systems in high-income countries (HIC), limiting their comparability with LMICs and emphasizing the need for more research in these countries [8–11].

Across Asian countries with lower-middle-income economies, Vietnam is a focal area for the rapid spread of drug-resistant infections [12]. Findings from the Vietnam Resistance Surveillance Network (VINARES) showed higher resistance rates (e.g., fourfold for ESBL and threefold for MRSA) in 2016–2017 compared to 2012–2013 [13]. These trends exemplified national health system challenges in addressing AMR [12,13]. In response, policymakers and key stakeholders have enacted AMR-relevant strategy documents and funding for interventions to prevent its emergence [12]. With support from global funding and the Vietnamese Ministry of Health, several surveillance platforms such as VINARES and Survey of Antibiotic Resistance (SOAR) have been set up to track the resistance prevalence of critical pathogens in hospitals [13]. However, these surveillance systems have not captured the economic cost of AMR, which cannot provide a comprehensive picture of changes in the burden of AMR over time for policy improvement. This limitation is particularly critical in the post-COVID pandemic in LMICs like Vietnam, where significant resource constraints and more pressing health priorities require careful allocation [14]. Therefore, despite multiple challenges in cost estimation, the economic impact of AMR in Vietnam needs to be largely investigated to set priorities for resource allocation.

Following the previous systematic reviews and published research, measuring hospital-based AMR burden could initiate the first steps in calculating AMR costs using localized data. This step allows for the gradual transformation of methodologies to other contexts and generating comparable estimates [10,11]. In Vietnamese hospitals, the increasing rates of carbapenem-resistant *Acinetobacter baumannii* (CRAB) and *Pseudomonas aeruginosa* (CRPA), third-generation cephalosporin-resistant *Escherichia coli* (3GCREC) and *Klebsiella pneumoniae* (3GCRKP), methicillin-resistant *Staphylococcus aureus* (MRSA) have been alarming [13]. Moreover, these pathogens have been highlighted in the World Health Organization (WHO) priority pathogen lists of 2017 and 2024, underscoring the critical need for research and novel treatments to combat these resistant infections [15]. Given the significant concerns of these pathogens in hospitals across Vietnam, this study aimed to estimate the incremental hospital costs and LOS associated with antimicrobial-resistant versus -susceptible among these pathogens from the healthcare sector perspective from one tertiary hospital in Vietnam from 2016 to 2021.

## Methods

### Study design

We conducted a retrospective observational study at the Hospital for Tropical Diseases in Ho Chi Minh City, Vietnam, from April 2016 to October 2021 to assess the incremental economic burden imposed by the priority pathogens as defined by WHO and Centers for Disease Control and Prevention (CDC) – CRAB, CRPA, 3GCREC, 3GCRKP, and MRSA. From the healthcare sector perspective, we estimated the incremental hospital costs incurred by inpatients with resistant infections versus those with susceptible infections. Besides, the effect of COVID-19 on the incremental hospital costs associated with resistant infections was evaluated by comparing the cost differences between the resistant and susceptible groups before and during COVID-19. The article was organized in alignment with the STROBE Statement [16].

### Study setting

The study hospital, with a bed capacity of 550, specializes in tropical infectious diseases for patients in southern Vietnam [17]. It also belongs to the VINARES network, which has a robust hospital information system for retrieving data on various patient characteristics [13].

### Study population

**Fig 1:**
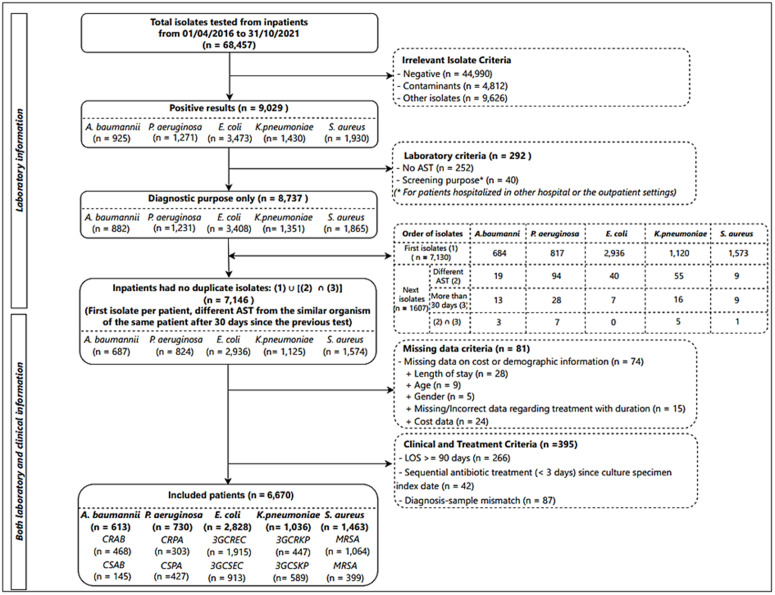
Flowchart of selecting patients for the study population.

We included patients hospitalized at the study hospital between April 1, 2016, and October 31, 2021, who had non-duplicate isolates from body locations for at least one of the following organisms: *A.baumannii, P.aeruginosa, E.coli, K.pneumoniae, and S.aureus*. To avoid duplicate isolates, we included only the first isolate of any species per 30-day episode for each patient during hospitalization. We also included isolates of the same species from the same patient if they showed different antimicrobial susceptibility profiles and were collected more than 30 days apart from the previous testing day [18]. Patients were required to receive sequential antibiotic treatments for at least three days since the index day when the culture specimen was obtained to minimize the likelihood of involving patients with colonization. We excluded patients with an extended LOS exceeding 90 days, as they might originate from other conditions, such as underlying chronic diseases, based on previous research studies and expert consultation [19,20]. Patients with clinical sample sites mismatched with primary diagnoses were also excluded as microorganisms like bacteria can be found on the skin, mucous membranes, open wounds, or body fluids without causing harmful clinical signs or symptoms [21]. The flowchart of selecting patients for the study population is shown in Fig 1.

### Variables and data collection

The data collected for each patient fell into three broad categories: outcomes, exposures, and covariates.

#### Outcomes.

The primary outcome was estimating the incremental hospital costs in patients with antibiotic-resistant pathogens versus those with susceptible ones. This was calculated as the mean difference in the hospital costs between patients with confirmed antibiotic-resistant pathogens and those with antibiotic-susceptible pathogens, with or without the covariate adjustments. From the healthcare sector perspective, the hospital costs included bed costs, doctor visits, medication costs (specializing in antibiotic costs), blood costs, testing costs, diagnostic costs, procedure costs, material costs, and transportation costs and did not include the subsequent costs that may arise following the initial hospitalization [22]. The costs were converted to 2023 US dollars using the purchasing power parity (PPP value = 8,239.36 VND/USD$ in 2023) and the Consumer Price Index (CPI) in 2016–2023 [23]. Besides, LOS was also included as a clinical outcome to evaluate resource use, hypothesizing that prolonged LOS would generate higher hospital costs. The secondary outcome was to measure the effect of COVID-19 on incremental hospital costs by estimating the differential cost between the resistant and susceptible groups of each pathogen at the COVID-19 occurrence point.

We chose June 2021 as the occurrence point of COVID-19 when the People’s Committee of Ho Chi Minh City implemented the social distancing levels under Government Directives 15, 16, and 17 (June-September 2021) before shifting to loosen the restrictions on October 1, 2021 [24,25]. Besides, following the expert consultation at the Hospital for Tropical Diseases in HCMC, although the loosened restrictions were enacted on October 1, 2021, the hospital still expanded the COVID-19 treatment until the end of October 2021 [26]. Therefore, we continued to collect the data up to October 2021.

#### Exposures.

According to the WHO pathogen list for guiding AMR prioritizing surveillance, there were five exposures that set values to five binary variables classified by causative bacteria and their susceptibility patterns in the study [15]:

- *A.baumanii* pathogens were grouped as carbapenem-resistant *A.baumannii* (CRAB) or carbapenem-susceptible *A.baumanii* (CSAB)
- *P.aeruginosa* pathogens were grouped as carbapenem-resistant *P.aeruginosa* (CRPA) or carbapenem-susceptible *P.aeruginosa* (CSPA)
- *E.coli* pathogens were classified as third-generation cephalosporin-resistant *E.coli* (3GCREC) or third-generation cephalosporin-susceptible *E.coli* (3GCSEC)
- *K.pneumoniae* pathogens were classified as third-generation cephalosporin-resistant *K.pneumoniae* (3GCRKP) or third-generation cephalosporin-susceptible *K.pneumoniae* (3GCSKP)
- *S.aureus* pathogens were classified as methicillin-resistant *S.aureus* (MRSA) or methicillin-susceptible *S.aureus* (MSSA)

The antimicrobial susceptibility testing results were obtained from the hospital microbiology laboratory by applying multiple techniques (disk diffusion, VITEK minimum inhibitory concentration, Etest strips) and standards of the Clinical and Laboratory Standards Institute (CLSI) reference [27]. All diagnostic tests were accredited by the external quality assurance scheme (UK-NEQAS). The laboratory’s final data on antimicrobial resistance was then submitted using WHONET Software (version 5.6) for data standardization and conducting multicenter research [13]. We labeled non-susceptible and intermediate susceptibility as resistant. Therefore, resistance to third-generation cephalosporins was defined as non-susceptible and intermediate susceptibility to ceftazidime and one of cefotaxime, ceftriaxone, or cefpodoxime. Similarly, some bacteria had non-susceptible and intermediate susceptibility results when testing meropenem and/or imipenem, and/or ertapenem would conclude as resistance to carbapenems. All MRSA isolates were defined as those *S.aureus* isolates resistant to oxacillin or cefoxitin. This definition represented that MRSA isolates were also resistant to all β-lactam antibiotics except for the anti-MRSA cephalosporins (ceftaroline) [28]. Furthermore, the microbiology laboratory disseminated the antibiotic susceptibility results through cascade reporting, meaning once the bacteria resistant to broad-spectrum antibiotic groups (such as carbapenems) are already resistant to other narrower antibiotic groups (such as penicillins and cephalosporins) [29].

#### Covariates.

All sociodemographic and clinical characteristics were obtained from patients’ electronic medical records, and each pathogen was estimated individually, stratified by antibiotic susceptibility results. We input variables that included sociodemographic characteristics of patients (age, sex, Body Mass Index (BMI), admission to ICU, health insurance beneft levels, location) and clinical characteristics (number of diagnoses, type of infection, specimen source site, Charlson comorbidity index (CCI), polymicrobial, LOS, treatment outcomes, antimicrobial groups prescribed) as adjusted covariates to estimate the incremental hospital costs per pathogen [30–32]. In this study, we also included the use of inpatient antibiotics during the entire hospitalization. However, as inpatients can use it before infections with five exposures, which are therefore not related to the infections, we consequently analyzed it for descriptive purposes only to limit the misleading conclusions.

### Statistical analysis

#### Descriptive statistics.

Statistical analyses were performed using R 4.3.0 and StataMP 17. Continuous variables normally distributed were reported as means (standard deviations), while continuous variables non-normally distributed were shown as medians (interquartile ranges (25^th^ and 75^th^ percentiles)). Student’s t-test was used to test mean differences, and the Mann-Whitney U test for median differences. Categorical variables were represented by counts (percentages), and intergroup differences were tested with the chi-squared or Fisher’s exact test for cell counts below 4. Our study set a p-value threshold of 0.05 for statistical significance.

#### Outcome estimates.

To examine the association of AMR with costs, generalized linear models (GLM) with a gamma distribution and log link were used to address the continuous, positive, and skewed characteristics of hospital cost data [33]. A multivariate generalized linear regression model alongside linear spline with one knot positioned at COVID-19 occurrence (June 2021) was employed to evaluate the incremental hospital costs associated with AMR with covariate adjustments. The covariates, including sociodemographic and clinical characteristics, showed the association in the literature review [30–32]. Our fully adjusted multivariate generalized linear regression model incorporated all sociodemographic, clinical variables, and an interaction term between CRAB and 3GCRKP. This combination of exposures accounted for the highest proportion of polymicrobial infection among patients with more than one isolate and demonstrated large main effects in the literature [34]. Similarly, given the over-dispersion of LOS data, we used a negative binomial regression model alongside a linear spline with one knot positioned at COVID-19 occurrence (June 2021) to predict LOS. Marginal effects were used to estimate the incremental hospital across five exposures in the study period. We applied McFadden pseudo R square, and the Akaike Information Criterion (AIC) and Bayesian Information Criterion (BIC) were employed to assess the fit of the GLM model.

#### The effect of COVID-19.

For examining the effect of COVID on the incremental hospital costs, the sample was stratified into two periods: before the COVID-19 pandemic (04/2016–05/2021) and during the peak of the COVID-19 pandemic’s fourth wave (during the COVID-19 for abbreviation) (06/2021–10/2021). Linear splines with one knot located at the COVID-19 occurrence (June 2021) were applied with the regression to evaluate the incremental hospital costs associated with AMR with adjustments for sociodemographic and clinical characteristics. Difference-in-difference (DiD) analysis was applied using marginal effects to estimate the differential cost between each pathogen’s resistant and susceptible groups regarding the COVID-19 pandemic. The results of the DiD analysis were presented as hospital costs change over time (before and during COVID-19) and between groups (resistant and susceptible groups for each specific pathogen). An adjusted DiD estimate, was determined by comparing the cost differences between the resistant and susceptible groups before and during COVID-19 and then subtracting these differences.

### Ethical consideration

All patient information was anonymized and stored as unlinked data before analysis to prevent personal information disclosure. The Ethics Committee in Biomedical Research at the Hospital for Tropical Diseases in HCMC approved this study proposal without requirements for participant consent under Decision 38/HDDD (September 30, 2019), Decision 1239/QD-BVBND (April 14, 2022), and Official Dispatch 775/BVBND-QLCL (April 22, 2024). Following this, the research data were accessed by April 22, 2024. Then, the final manuscript was reviewed and accepted for journal submission by the Hospital through the Official Dispatch 2812/BVBND-QLCL (October 23, 2024).

## Results

### Sociodemographic and clinical characteristics of the patients

During the six-year period, of the 6,670 patients identified through the inclusion criteria, 4,197 patients (62.92%) were detected as priority pathogens, with 1915 (45.63%) and 1064 (25.33%) due to 3GCREC and MRSA, respectively. Among patients with *A. baumannii* isolates, 76.35% (468 patients) had carbapenem-resistant strains, while among patients with *S. aureus* isolates, 72.73% (1064 patients) developed methicillin resistance. The distribution of patients by sociodemographic characteristics for each pathogen is illustrated in Table 1 and S1 Table. Most patients were adults aged older than 50 years old, except for patients with *S.aureus* isolates with a median age ranging from 35–38 years old (38 in MRSA vs 35 in MSSA; p-value = 0.36). There were 212 (45.30%) patients with CRAB isolates and 188 (42.06%) patients who had 3GCRKP isolates who did not participate in the health insurance program, remarkably higher than those with CSAB and 3GCSKP isolates.

**Table 1:** Sociodemographic characteristics of patients for each pathogen individually with stratification by five exposures.

Characteristics	*A.baumannii*		*P. aeruginosa*		*E.coli*		*K.pneumoniae*		*S. aureus*	
	CRAB (N = 468)	CSAB (N = 145)	CRPA (N = 303)	CSPA (N = 427)	3GCREC (N = 1915)	3GCSEC (N = 913)	3GCRKP (N = 447)	3GCSKP (N = 589)	MRSA (N = 1064)	MSSA (N = 399)
**Gender (n, %)**										
Male	279 (59.61%)	86 (59.31%)	195 (64.36%)	283 (66.28%)	705 (36.81%)	249 (27.27%)	222 (49.66%)	396 (67.23%)	726 (68.23%)	275 (68.92%)
Female	189 (40.39%)	59 (40.69%)	108 (35.64%)	144 (33.72%)	1,210 (63.19%)	664 (72.73%)	225 (50.34%)	193 (32.77%)	338 (31.77%)	124 (31.08%)
**Age, median (Q1-Q3)**	58 (41, 69)	37 (22, 56)	59 (41, 70)	50 (33, 66)	56 (41, 67)	52 (38, 65)	56 (40, 69)	52 (42,62)	38 (23, 54)	35 (23, 52)
**Living in Ho Chi Minh City, n (%)**	253 (54.06%)	57 (39.31%)	146 (48.18%)	150 (35.13%)	835 (43.60%)	495 (54.22%)	238 (53.24%)	273 (46.35%)	491 (46.14%)	194 (48.62%)
**Admission to ICU, n (%)**	245 (52.35%)	51 (35.17%)	175 (57.76%)	210 (49.18%)	228 (11.91%)	61 (6.68%)	190 (42.51%)	134 (22.75%)	183 (17.20%)	74 (18.55%)
**Health insurance benefit levels, (n, %)**										
0%	212 (45.30%)	38 (26.21%)	109 (35.98%)	106 (24.82%)	505 (26.37%)	276 (30.23%)	188 (42.06%)	157 (26.66%)	292 (27.44%)	122 (30.57%)
80%	142 (30.34%)	56 (38.62%)	99 (32.67%)	180 (42.15%)	977 (51.02%)	497 (54.44%)	140 (31.32%)	302 (51.27%)	488 (45.86%)	175 (43.86%)
95%	9 (1.92%)	2 (1.38%)	11 (3.63%)	16 (3.75%)	74 (3.86%)	19 (2.08%)	7 (1.57%)	28 (4.75%)	22 (2.07%)	9 (2.26%)
100%	105 (22.44%)	49 (33.79%)	84 (27.72%)	125 (29.28%)	359 (18.75%)	121 (13.25%)	112 (25.05%)	102 (17.32%)	262 (24.62%)	93 (23.31%)

Gray shading indicates statistically significant hypothesis testing results (usually p < 0.05) from the chi-squared or Fisher’s exact test (for categorical data) and the Mann-Whitney U test (for non-parametric data)

For clinical characteristics, the median specimen collection date to identify CRPA was 19, with an interquartile range between 11 and 29, in contrast to MRSA and 3GCREC, which were often taken from 0 to 3 days for pathogen detection (Table 2). The CCI of most patients (75% − 80% depending on pathogen type) was 0, showing the similarity of the comorbidity burden among these patients. Patients with CRPA had significantly prolonged LOS compared to CSPA (median, 42 vs 33 days, p-value < 0.001). Regarding treatment outcomes, patients having CRAB pathogens exhibited worse prognoses (Unchanged, Deteriorated, or Died) at 60.90%. In contrast, most patients with other pathogens recovered after treatment, with improvement rates ranging from 48.51% for CRPA to 85.22% for 3GCREC.

**Table 2:** Clinical characteristics of patients for each pathogen individually with stratification by five exposures.

Characteristics	*A.baumannii*		*P. aeruginosa*		*E.coli*		*K.pneumoniae*		*S. aureus*	
	CRAB (N = 468)	CSAB (N = 145)	CRPA (N = 303)	CSPA (N = 427)	3GCREC (N = 1915)	3GCSEC (N = 913)	3GCRKP (N = 447)	3GCSKP (N = 589)	MRSA (N = 1064)	MSSA (N = 399)
**Specimen collection date** ^**(c)**^	11 (6, 18)	6 (1, 14)	19 (11, 29)	10 (2, 18)	0 (0, 2)	0 (0, 1)	9 (1,16)	1 (0, 6)	0 (0, 3)	0 (0, 2)
**Charlson comorbidity index (CCI)**										
0	354 (75.64%)	117 (80.69%)	230 (75.91%)	344 (80.56%)	1,468 (76.66%)	681 (74.59%)	340 (76.06%)	380 (64.52%)	861 (80.92%)	332 (83.21%)
1	21 (4.49%)	2 (1.38%)	14 (4.62%)	14 (3.28%)	34 (1.78%)	10 (1.10%)	20 (4.47%)	5 (0.85%)	17 (1.60%)	9 (2.26%)
2	53 (11.32%)	15 (10.34%)	29 (9.57%)	35 (8.20%)	311 (16.24%)	177 (19.39%)	54 (12.08%)	174 (29.54%)	93 (8.74%)	23 (5.76%)
≥3	40 (8.55%)	11 (7.59%)	30 (9.90%)	34 (7.96%)	102 (5.33%)	45 (4.93%)	33 (7.38%)	30 (5.09%)	93 (8.74%)	35 (8.77%)
**Type Of Infection**										
Bloodstream Infection	72 (15.38%)	24 (16.55%)	39 (12.87%)	57 (13.35%)	810 (42.30%)	460 (50.38%)	80 (17.90%)	259 (43.97%)	241 (22.65%)	95 (23.81%)
COVID-19	164 (35.04%)	12 (8.28%)	67 (22.11%)	26 (6.09%)	15 (0.78%)	5 (0.55%)	121 (27.07%)	21 (3.57%)	23 (2.16%)	2 (0.50%)
Lower respiratory tract infection (LRTI)	24 (5.13%)	14 (9.66%)	22 (7.26%)	33 (7.73%)	36 (1.88%)	23 (2.52%)	14 (3.13%)	7 (1.19%)	35 (3.29%)	3 (0.75%)
Intra-abdominal infection	8 (1.71%)	3 (2.07%)	6 (1.98%)	5 (1.17%)	132 (6.89%)	87 (9.53%)	17 (3.80%)	72 (12.22%)	11 (1.03%)	9 (2.26%)
Meningitis	20 (4.27%)	6 (4.14%)	16 (5.28%)	20 (4.68%)	41 (2.14%)	17 (1.86%)	16 (3.58%)	16 (2.72%)	16 (1.50%)	16 (4.01%)
Skin Infection	3 (0.64%)	2 (1.38%)	8 (2.64%)	21 (4.92%)	8 (0.42%)	6 (0.66%)	2 (0.45%)	6 (1.02%)	318 (29.89%)	96 (24.06%)
Urinary tract infection	3 (0.64%)	10 (6.90%)	1 (0.33%)	5 (1.17%)	426 (22.25%)	162 (17.74%)	31 (6.94%)	25 (4.24%)	9 (0.85%)	3 (0.75%)
Tetanus	84 (17.95%)	29 (20.00%)	70 (23.10%)	144 (33.72%)	87 (4.54%)	26 (2.85%)	42 (9.40%)	94 (15.96%)	104 (9.77%)	43 (10.78%)
HIV	17 (3.63%)	10 (6.90%)	11 (3.63%)	26 (6.09%)	146 (7.62%)	42 (4.60%)	36 (8.05%)	31 (5.26%)	167 (15.70%)	58 (14.54%)
Other Diseases (*)	73 (15.60%)	35 (24.14%)	63 (20.79%)	90 (21.08%)	214 (11.17%)	85 (9.31%)	88 (19.69%)	58 (9.85%)	140 (13.16%)	74 (18.55%)
**Treatment Outcomes**										
Improved	183 (39.10%)	101 (69.66%)	147 (48.51%)	284 (66.51%)	1,632 (85.22%)	794 (86.97%)	231 (51.68%)	432 (73.34%)	834 (78.38%)	322 (80.70%)
Unchanged	67 (14.32%)	17 (11.72%)	42 (13.86%)	59 (13.82%)	157 (8.20%)	61 (6.68%)	59 (13.20%)	76 (12.90%)	138 (12.97%)	45 (11.28%)
Worsen	86 (18.38%)	18 (12.41%)	69 (22.77%)	69 (16.16%)	114 (5.95%)	58 (6.35%)	73 (16.33%)	69 (11.71%)	73 (6.86%)	29 (7.27%)
Deceased	132 (28.21%)	9 (6.21%)	45 (14.85%)	15 (3.51%)	12 (0.63%)	0 (0.00%)	84 (18.79%)	12 (2.04%)	19 (1.79%)	3 (0.75%)
**Length of stay (LOS), median (Q1-Q3)**	29 (18, 42)	24 (13, 39)	42 (28, 56)	33 (17, 46)	11 (8, 15)	9 (8, 12)	24 (14, 39)	16 (10, 30)	14 (8, 25)	12 (8, 25)

Notes: (*) Other diseases, including infectious diseases accounting for small proportions such as upper respiratory infection and chronic diseases (hypertension, diabetes,...);

Gray shading indicates statistically significant hypothesis testing results (usually p < 0.05) from the chi-squared or Fisher’s exact test (for categorical data) and the Mann-Whitney U test (for non-parametric data)

### Estimation of hospital cost

From the median unadjusted cost data shown in S2 Fig, most patients with antibiotic-resistant pathogens had significantly higher hospital costs than those with antibiotic-susceptible pathogens (p-value < 0.001), except for MRSA vs. MSSA (median, $1,471 vs $1,520, p-value = 0.35). Medication cost (specifically antibiotic cost) and procedure cost made a significant contribution (between 54,0% and 77,0%) to the difference between antibiotic-resistant pathogens and antibiotic-susceptible ones.

After adjusting for various sociodemographic and clinical characteristics, antibiotic-resistant pathogens (five exposures) remained robust predictors of hospital costs (Table 3). In the fully adjusted model, the incremental hospital cost was highest among one patient isolated with CRAB pathogen compared to one with CSAB pathogen (marginal cost, $3,980 (95%CI: $3,170 - $4,790)), followed by the incremental hospital cost associated with 3GCRKP versus 3GCSKP pathogens (marginal cost $1,942 (95%CI: $1,212 - $2,672)). Conversely, one patient tested with MRSA result paid lower hospital costs (-$326 (95%CI: -$806 - -$154) than one with MSSA. Male patients, participation in health insurance with higher benefit level, ICU admission, higher CCIs, worse prognosis, or those who had prolonged LOS were likely to pay higher hospital costs (S3 Table).

**Table 3:** Incremental hospital costs of AMR-related inpatients by antibiotic-resistant pathogens (five exposures).

Comparisons	Model 1 Marginal effect (95%CI) ($,2023)	Model 2 Marginal effect (95%CI) ($,2023)	Model 3 Marginal effect (95%CI) ($,2023)	Model 4 Marginal effect (95%CI) ($,2023)	Model 5 Marginal effect (95%CI) ($,2023)
*CRAB vs. CSAB*	17,041*** (15,068 − 19, 015)	13,362*** (11,822 − 14,902)	7,645*** (6,576 − 8,714)	5,576*** (4,561 − 6,592)	3,980*** (3,170 − 4,790)
*CRPA vs. CSPA*	20,368*** (16,998 − 23,737)	17,337*** (14,408 − 20,266)	10,628*** (8,056 – 13,200)	997* (167 − 1,827)	1,000* (225 − 1,775)
*3GCREC vs. 3GCSEC*	−490 (−1,513 - 533)	447 (−422 − 1,316)	461 (−279 − 1,200)	195 (−298 - 688)	444* (6 - 894)
*3GCRKP vs. 3GCSKP*	15,474*** (13,090 − 17,857)	10,994*** (9,241 − 12,747)	5,877*** (4,843 − 6,912)	2,740*** (1,911 − 3,568)	1,942*** (1,212 − 2,672)
*MRSA vs. MSSA*	1,444** (446 − 2,442)	2,295*** (1,208 − 3,381)	1,306*** (537 − 2,074)	−485 (−1,037 - 68)	−326* (−806 - −154)

Note: Model 1: unadjusted generalized linear model. Model 2, 3, 4, 5: linear splines with one knot in June 2021 and generalized linear regression models. Model 2: adjusted for age and sex; Model 3: adjusted for sociodemographic variables (gender, age, health insurance benefit levels, admission to ICU). Model 4: adjusted for age, sex, and clinical variables (type of infection, Charlson Comorbidity Index, LOS, treatment outcomes). Model 5: a fully adjusted model for all sociodemographic and clinical variables and the interaction between *A.baumannii* and *K.pneumoniae*. * p < 0.05, ** p < 0.01, *** p < 0.001. CI – Confidence interval

### Incremental length of stay

Table 4 presents the incremental LOS when comparing one patient in the resistant group to one in the susceptible group. In the fully adjusted model, all patients with resistance exposure had significantly prolonged LOS compared to patients in the susceptible group. The incremental length of stay was highest among one patient isolated with CRPA vs. CSPA (12.54 days, 95%CI: 11.12–13.97 days), while the lowest for each patient with 3GCREC vs 3GCSEC (1.40 days, 95%CI: 0.69–2.10 days).

**Table 4:** Incremental length of stay of AMR-related inpatients by antibiotic-resistant pathogens (five exposures).

Comparisons	Model 1 Marginal effect (95%CI) (days)	Model 2 Marginal effect (95%CI) (days)	Model 3 Marginal effect (95%CI) (days)	Model 4 Marginal effect (95%CI) (days)	Model 5 Marginal effect (95%CI) (days)
*CRAB vs. CSAB*	12.31*** (10.99 - 13.63)	12.43*** (10.99 - 13.87)	7.00*** (5.80 - 8.19)	8.65*** (7.47 - 9.82)	7.07*** (5.90 - 8.23)
*CRPA vs. CSPA*	22.50*** (20.74 - 24.25)	20.92*** (19.14 - 22.69)	14.06*** (12.68 - 15.44)	14.00*** (12.43 - 15.57)	12.54*** (11.12 - 13.97)
*3GCREC vs. 3GCSEC*	0.24 (−0.63 - 1.10)	0.94* (0.04 - 1.84)	0.90* (0.13 - 1.68)	1.63*** (0.86 - 2.39)	1.40*** (0.69 - 2.10)
*3GCRKP vs. 3GCSKP*	10.12*** (8.70 - 11.54)	10.09*** (8.62 - 11.56)	6.51*** (5.28 - 7.74)	7.97*** (6.71 - 9.23)	6.80*** (5.57 - 8.04)
*MRSA vs. MSSA*	5.23*** (4.14 - 6.32)	5.04*** (3.94 - 6.14)	4.77*** (3.82 - 5.72)	5.06*** (4.06 - 6.06)	4.96*** (4.02 - 5.91)

Note: Model 1: unadjusted negative binomial regression model. Model 2, 3, 4, 5: linear splines with one knot in June 2021 and negative binomial regression models. Model 2: adjusted for age and sex, Model 3: adjusted for sociodemographic variables (gender, age, health insurance benefit levels, admission to ICU). Model 4: adjusted for age, sex, and clinical variables (type of infection, CCI, treatment outcomes). Model 5: a fully adjusted model for all sociodemographic and clinical variables and the interaction between *A.baumannii* and *K.pneumoniae*. * p < 0.05, ** p < 0.01, *** p < 0.001. CI – Confidence interval

### The effect of COVID-19 pandemic

**Fig 2:**
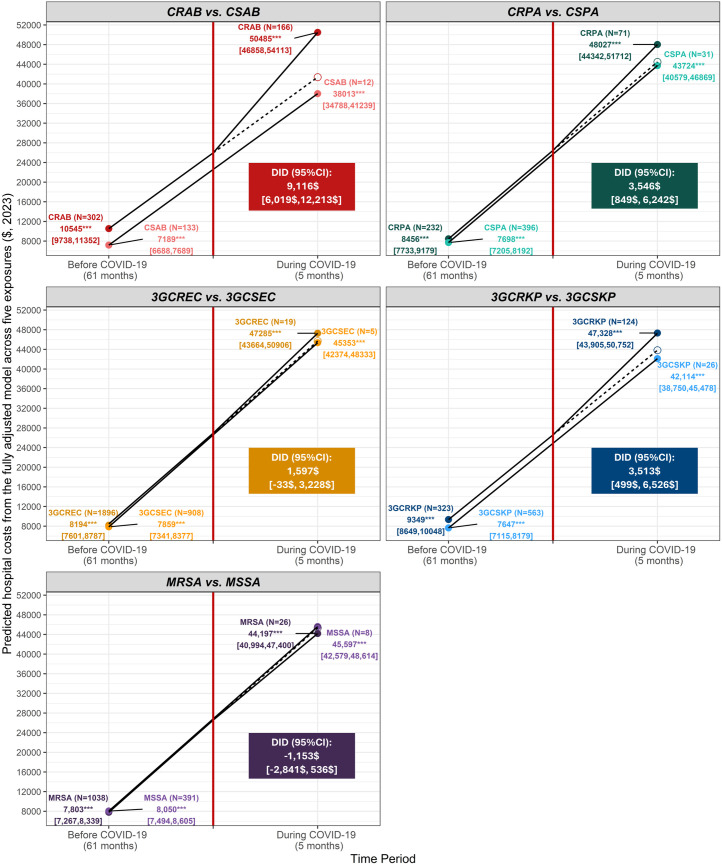
The effect of COVID-19 on incremental hospital costs associated with AMR.

Fig 2 illustrates the DiD analysis, focusing on the effect of COVID-19 on hospital costs among different pathogens with marginal effect estimation. While accounting for covariates, we found that the hospital costs of both patients having resistant or susceptible pathogens increased sharply during COVID-19 compared to before COVID-19. Specifically, the hospital costs among patients having 3GCRKP and 3GCSKP isolates between two periods raised by $37,979 and $34,467, respectively. From the fully adjusted model, the DiD estimate showed that the differences in the hospital costs between resistant and susceptible profiles for *A.baumannii* (CRAB vs. CSAB), *P.aeruginosa* (CRPA vs. CSPA), *K.pneumoniae* (3GCRKP vs. 3GCSKP) significantly widened during COVID-19. Among the three mentioned pathogens, the most significant difference was observed in *A.baumannii* with the DiD estimate of $9,116 (95%CI: $6,019 - $12,213).

## Discussion

To our knowledge, this is the first study estimating the costs of AMR in Vietnam and LMIC from the healthcare sector perspective. From 2016 to 2021, 4,197 out of 6,670 patients (62.92%) were isolated with priority pathogens, with the highest prevalence of priority pathogens observed in 3GCREC and MRSA (accounting for 45.63% and 25.33%, respectively). After adjusting covariates by regression modeling, we found that the correspondingly adjusted incremental hospital cost of one patient in the resistant group was significantly higher except for patients tested with MRSA results (average, CRAB, CRPA, 3GCREC 3GCRKP, MRSA were 3,980, 1,000, 444, 1,942, and −326 in $, 2023, respectively). The incremental LOS ranged from 1.40 days (95%CI: 0.69–2.10) for 3GCREC vs. 3GCSEC patients to 12.54 days (95%CI: 11.12–13.97 ) for CRPA vs. CSPA patients. The DiD estimate, considering covariates, revealed that COVID-19 significantly enlarged the hospital cost gaps between patients with resistant and susceptible profiles for *A.baumannii* (CRAB vs. CSAB), *P.aeruginosa* (CRPA vs. CSPA), and *K.pneumoniae* (3GCRKP vs. 3GCSKP). Among the pathogens, *A.baumannii* showed the highest difference at $9,116 (95%CI: $6,019 - $12,213).

Given the heavy financial pressure imposed by the WHO bacterial priority pathogens, including ESKAPE ones, one systematic review of the economic burden of AMR (2023) [7] showed that the attributable costs of AMR ranged from $9,516.5 ($, 2020 – China) to $12,043.5 ($, 2020 – China) for CRAB. Additionally, the attributable costs for MRSA varied from -$2,371.40 ($, 2020) in the USA to $1,627.60 ($, 2020) in Japan [7]. Our findings on incremental hospital costs of patients with antibiotic-resistant were in accordance with those studies in middle-income countries (MIC) (China, Lebanon, Thailand), indicating that hospital costs were quite similar in MIC settings and much lower than those in high-income countries (HIC) [7]. This might be explained by less advanced treatment options, lower per-unit resource costs, and exchange rate differences in MIC settings [7]. Hospitals in Vietnam may discharge patients earlier due to limited bed capacity and high patient volumes, which affects ongoing monitoring and comprehensive treatment to track the patient’s status [35]. Besides, when broken down into cost components, the bed day price is relatively low in Vietnam as well as compared to other developed countries, at approximately 30.99$ (medical bed) – 105.29$ (ICU bed) per day [32,36]. Regarding medication costs, the Vietnamese predominantly use generic pharmaceuticals, and medications might be procured at lower prices through a centralized drug procurement process by the public sector and international aid programs [37]. As bed costs and medication costs were significant contributory cost components to the total hospital cost, lower bed day and medication costs resulted in lower total hospital cost.

Compared with patients with susceptible Gram-negative pathogens, those with resistance had significantly increased hospital costs and prolonged LOS. For Gram-negative infection, our study also indicated that CRAB and 3GCRKP infections require more expensive treatments than other infections, with the adjusted cost difference per patient of $3,980 and $1,942 compared to CSAB and 3GCSKP, respectively. Results from earlier studies [38,39] also demonstrated similar comparisons in which CRAB and 3GCRKP patients had to pay more than CRPA and 3GCREC ones. Findings from one multicenter study in China also indicated that treatment of CRAB infections incurred a higher total hospital cost ($7,277) compared to CRPA ($4,605) [38]. CRAB often causes moderate-to-severe infection and leads to limited treatment options, which require combinations of antibiotics and expensive last-resort antibiotics (such as colistin, tigecycline, and fosfomycin) [40]. Besides, therapeutic drug monitoring costs alongside the drug costs may significantly drive up the total hospital costs. Furthermore, there is a need for infection control measures targeting CRAB and 3GCRKP within hospital settings and antibiotic stewardship programs (ASPs) to reduce the prevalence and economic burden of these infections. This finding has taken a preliminary step to set priorities for addressing the financial impact of CRAB and 3GCRKP infections. Future research should delve deeper into differentiating between invasive and non-invasive infections caused by CRAB and 3GCRKP to target better infection-prevention efforts, economic planning, and cross-setting comparison.

While the CRAB pathogen showed the association with the highest incremental hospital costs, CRPA drove the most prolonged LOS among pathogens. Regarding LOS results, studies show varied findings when comparing the prolonged LOS between patients with CRAB and CRPA infections. Zhen (2020) reported that CRAB was associated with a longer excess LOS (15.8 days) compared to CRPA (5.4 days) [38]. However, another study in the United States indicated that CRPA was associated with an inpatient post-culture LOS of 27 days, which was slightly longer than CRAB at 26 days [41]. Our analysis measured the total LOS, including pre-exposure and post-exposure LOS. While this comprehensive approach to estimating the total LOS captured the overall burden, it might obscure differences in the direct impact of each pathogen and align with previous research. Further research should investigate the analysis using only post-exposure LOS to clarify pathogen-specific differences more precisely.

Considering that MRSA was predicted to have the highest AMR-related mortality, morbidity, and healthcare costs in the Western Pacific Region in the next decade [42], our study found that the MRSA treatment was less expensive than the MSSA one. This finding also supports the conclusions drawn by Klein et al [43]. Based on a literature review [43], the first explanation might stem from the type of infection caused by the MRSA/MSSA isolates. Our results of patient characteristics showed that a high proportion of MRSA patients had non-invasive skin and soft tissue infections, indicated with second-line drugs to make cost savings. In contrast, patients diagnosed with invasive infections (such as bloodstream infections, LRTI) caused by MSSA were prescribed anti-staphylococcal penicillin, which has much higher drug costs than vancomycin [43]. Besides, the increased use of empirical vancomycin may lead to earlier optimal therapy for MRSA patients, thereby improving outcomes and reducing costs. Our study showed that about 60.43% of MRSA patients and 34.59% of MSSA patients were treated with vancomycin (part of the Glycopeptide antibacterials (J01XA) group). However, studies by McDanel et al [44] showed that prescribing empirical vancomycin treatment for patients with MSSA might also reduce the effectiveness of sequential therapy with anti-staphylococcal penicillin and cefazolin and worse outcomes. In addition, stringent infection control measures for MRSA at the hospital also reduce the average treatment costs of MRSA patients compared to MSSA patients. Specifically, hospitals performed less invasive diagnostic testing and empirical treatment of MRSA cases, increasing the number of cases diagnosed with this infection in the electronic system data while reducing the occurrence of cross-infection in the hospital setting.

When assessing the effect of COVID-19 on the economic burden of AMR pathogens using DiD analyses, the study noted that the gaps between resistant and susceptible cases were broadened during the COVID-19 pandemic. Several factors have partially contributed to this widening gap, including advanced infection control measures, disruptions in routine healthcare services, and changes in antibiotic prescribing practices [45]. During the COVID-19 epidemic, as the study hospital was one of the frontline hospitals for treating COVID-19 patients, the patients remaining in the hospital primarily had underlying, complex conditions requiring diligent monitoring by healthcare staff [46]. Moreover, heightened antibiotic use aimed at reducing short-term COVID-19 mortality might not only have driven up hospital costs for patients with susceptible or resistant results. This practice also increased long-term AMR-related mortality due to the development of multidrug-resistant bacteria and cross-infection during the pandemic [47]. Therefore, the key lesson from COVID-19 in response to future pandemic scenarios is the need to enhance infection control post-pandemic to limit multidrug-resistant bacteria and promptly develop the standard procedures for drug utilization when an outbreak occurs. In particular, antibiotic use management needs to be tightened after the pandemic because doctors’ treatment habits may not change immediately [48]. On the other hand, in the period 2016–2021, besides the effect of COVID-19, there were also ASP-related policy changes in June 2018 that might have indirectly impacted the costs associated with AMR [49,50]. Our study examined the effect of both policy change and COVID-19 on the incremental hospital costs by employing a multivariate generalized linear regression model alongside splines with two knots positioned at ASP-related policy change (June 2018) and COVID-19 occurrence (June 2021). However, this analysis showed a high correlation between pre- and post-ASP-related policy change periods, indicating that this change had almost no or gradual impact on the incremental hospital costs associated with AMR. This might be due to the hospital’s focus on strengthening the antimicrobial use and AMR surveillance system in the early stage of ASP before the next objective of understanding the actual burden of resistant infections. Therefore, we did not add this ASP-related policy change knot to the final model.

This study has some strengths regarding novel algorithms in cost estimation and data collection sources. Firstly, although this study was conducted in one tertiary hospital, this study hospital specializing in infectious diseases, accompanied by long-term data collection and highly relevant clinical data sources (such as microbiology data), might enhance the study’s reliability and analytical depth. Currently, estimating the actual economic burden of AMR in LMICs remains challenging due to the lack of effective antimicrobial resistance surveillance systems and the cost data reported by different types of infection rather than their infectious agents [51,52]. For example, hospital data systems will record the cost of treating bacteremia, skin infections, etc., instead of MRSA treatment costs [52]. The absence of comprehensive economic calculations for AMR infection might lead to underestimating their actual impact, hindering proper assessment and management of AMR burden, especially in resource-constrained LMIC settings [52]. Secondly, we subtracted the mean cost of antimicrobial-susceptible infections from those with antimicrobial-resistant infections for incremental hospital cost estimation associated with AMR rather than using uninfected cases as controls to prevent exaggerating its economic impact. Thirdly, by incorporating splines into the regression model, the novel algorithm effectively captured the effect of interventions (such as policy shifts or COVID-19) on incremental AMR-associated hospital costs, offering a more precise analysis and fostering a better understanding and support for policy measures.

One limitation of this study is its retrospective observational design at a single tertiary hospital, which may introduce biases and prevent the collection of certain variables for insight and comparison purposes, like community-acquired versus hospital-acquired infections. Therefore, in the future, we will re-implement the study prospectively in some hospitals to strengthen the findings. Additionally, our regression model accounted only for observable covariates, which might leave some unobservable biases unaddressed. Nevertheless, the covariates for hospital cost adjustments were based on a literature review, providing valuable insights into cost drivers [30–32]. Besides, as COVID-19 transitions from pandemic to endemic with conflicting evidence about its end time in Vietnam, we evaluated only the short-term effect during the fourth wave’s peak (End of May-October 2021). Therefore, it might lead to a small sample size during this period and overestimation results [53]. Finally, our assessment was confined to in-hospital medical costs, not including post-discharge costs or societal impacts such as productivity losses, which could not reflect the actual economic impact of AMR.

## Conclusions

From the healthcare sector perspective, resistant infections caused by five priority pathogens were associated with higher hospital costs among inpatients. These estimates could provide valuable insights for policymakers to develop strategies and set priorities for resource allocation. Future research should investigate detailed analyses (for example, differentiate between invasive and non-invasive infections caused by CRAB and 3GCRKP) for specifically tailoring infection-prevention efforts.

## Supporting information

S1 Table Characteristics of the sample. (PDF)

S2 Fig Unadjusted cost estimates by components. (TIF)

S3 Table Results of multivariate generalized linear regression. (PDF)

S4 Table Results of multivariate negative binomial regression. (PDF)

## Abbreviations

AMR: Antimicrobial resistance
LOS: Length of stay
GDP: Gross Domestic Product
LMIC: Low- and Middle-Income Country
MIC: Middle-Income Country
HIC: High-Income Country
VINARES: Vietnam Resistance Surveillance Network
ESKAPE: *Enterococcus faecium, Staphylococcus aureus, Klebsiella pneumoniae, Acinetobacter baumannii, Pseudomonas aeruginosa*, and *Enterobacter species*
CRAB: Carbapenem-resistant *Acinetobacter baumannii*
CSAB: Carbapenem-susceptible *Acinetobacter baumannii*
CRPA: Carbapenem-resistant *Pseudomonas aeruginosa*
CSPA: Carbapenem-susceptible *Pseudomonas aeruginosa*
3GCREC: Third-generation cephalosporin-resistant *Escherichia coli*
3GCSEC: Third-generation cephalosporin-susceptible *Escherichia coli*
3GCRKP: Third-generation cephalosporin-resistant *Klebsiella pneumoniae*
3GCSKP: Third-generation cephalosporin-susceptible *Klebsiella pneumoniae*
MRSA: methicillin-resistant *Staphylococcus aureus*
MSSA: methicillin-susceptible *Staphylococcus aureus*
ICU: Intensive care unit
CCI: Charlson Comorbidity Index
ASP: Antibiotic Stewardship Program.
