# Supplementary material for: Healthcare utilization associated with antimicrobial resistance at a tertiary hospital in Vietnam: A retrospective observational study from 2016 to 2021
Source: PLoS One. 2025 Aug 4;20(8):e0329539. doi: 10.1371/journal.pone.0329539 (PMC12321119; doi:10.1371/journal.pone.0329539)
Supplement: S1 Table — (PDF) [file pone.0329539.s001.pdf]

S1 Table. Characteristics of the sample

Characteristics	*Acinetobacter baumannii*		*Pseudomonas aeruginosa*		*Escherichia coli*		*Klebsiella pneumoniae*		*Staphylococcus aureus*	
	**CRAB**	**CSAB**	**CRPA**	**CSPA**	**3GCREC**	**3GCSEC**	**3GCRKP**	**3GCSKP**	**MRSA**	**MSSA**
**Number of inpatients, n**	468	145	303	427	1915	913	447	589	1064	399
**Body Mass Index (BMI), (n, %)**										
Underweight	68 (14.53%)	37 (25.52%)	47 (15.51%)	110 (25.76%)	321 (16.76%)	148 (16.21%)	76 (17.00%)	84 (14.26%)	301 (28.29%)	135 (33.83%)
Normal	258 (55.13%)	70 (48.28%)	176 (58.09%)	222 (51.99%)	1,129 (58.96%)	554 (60.68%)	261 (58.39%)	360 (61.12%)	588 (55.26%)	207 (51.88%)
Overweight	108 (23.08%)	29 (20.00%)	63 (20.79%)	65 (15.22%)	379 (19.79%)	173 (18.95%)	81 (18.12%)	111 (18.85%)	136 (12.78%)	37 (9.27%)
Obesity	34 (7.26%)	9 (6.21%)	17 (5.61%)	30 (7.03%)	86 (4.49%)	38 (4.16%)	29 (6.49%)	34 (5.77%)	39 (3.67%)	20 (5.01%)
p-value [(a)]	0.024		0.004		0.85		0.61		0.047	
**Year of hospitalization**										
2016	30 (6.41%)	20 (13.79%)	33 (10.89%)	40 (9.37%)	375 (19.58%)	158 (17.31%)	27 (6.04%)	88 (14.94%)	131 (12.31%)	52 (13.03%)
2017	59 (12.61%)	38 (26.21%)	58 (19.14%)	60 (14.05%)	396 (20.68%)	206 (22.56%)	56 (12.53%)	116 (19.69%)	195 (18.33%)	80 (20.05%)
2018	56 (11.97%)	32 (22.07%)	29 (9.57%)	78 (18.27%)	320 (16.71%)	140 (15.33%)	67 (14.99%)	79 (13.41%)	175 (16.45%)	67 (16.79%)
2019	68 (14.53%)	18 (12.41%)	46 (15.18%)	98 (22.95%)	410 (21.41%)	170 (18.62%)	68 (15.21%)	114 (19.35%)	243 (22.84%)	95 (23.81%)
2020	65 (13.89%)	18 (12.41%)	50 (16.50%)	86 (20.14%)	295 (15.40%)	157 (17.20%)	80 (17.90%)	118 (20.03%)	216 (20.30%)	66 (16.54%)
2021	190 (40.60%)	19 (13.10%)	87 (28.71%)	65 (15.22%)	119 (6.21%)	82 (8.98%)	149 (33.33%)	74 (12.56%)	104 (9.77%)	39 (9.77%)
p-value [(a)]	<0.001		<0.001		0.018		<0.001		0.72	
**Comparison to the registered hospital's level, n(%)**										
No comparison	212 (45.30%)	38 (26.21%)	109 (35.97%)	106 (24.82%)	505 (26.37%)	276 (30.23%)	188 (42.06%)	157 (26.66%)	292 (27.44%)	122 (30.58%)
At level different from the registered hospital	18 (3.85%)	28 (19.31%)	11 (3.63%)	41 (9.60%)	764 (39.90%)	360 (39.43%)	69 (15.44%)	143 (24.28%)	338 (31.77%)	122 (30.58%)
Same level as the registered hospital	238 (50.85%)	79 (54.48%)	183 (60.40%)	280 (65.57%)	646 (33.73%)	277 (30.34%)	190 (42.51%)	289 (49.07%)	434 (40.79%)	155 (38.85%)
p-value [(a)]	<0.001		0.062		<0.001		<0.001		0.49	
**Specimen source site, n(%)**										
BAL fluid	359 (76.71%)	69 (47.59%)	206 (67.99%)	212 (49.65%)	28 (1.46%)	6 (0.66%)	184 (41.16%)	113 (19.19%)	116 (10.90%)	48 (12.03%)
Blood	41 (8.76%)	20 (13.79%)	14 (4.62%)	40 (9.37%)	501 (26.16%)	341 (37.35%)	57 (12.75%)	252 (42.78%)	214 (20.11%)	104 (26.07%)
Urine	28 (5.98%)	35 (24.14%)	39 (12.87%)	52 (12.18%)	1,210 (63.19%)	443 (48.52%)	151 (33.78%)	97 (16.47%)	28 (2.63%)	14 (3.51%)
Wound	15 (3.21%)	7 (4.83%)	17 (5.61%)	70 (16.39%)	18 (0.94%)	8 (0.88%)	19 (4.25%)	22 (3.74%)	506 (47.56%)	161 (40.35%)
Peritoneal fluid	2 (0.43%)	3 (2.07%)	2 (0.66%)	0 (0.00%)	121 (6.32%)	97 (10.62%)	13 (2.91%)	68 (11.54%)	3 (0.28%)	2 (0.50%)
Pus	1 (0.21%)	2 (1.38%)	3 (0.99%)	16 (3.75%)	5 (0.26%)	2 (0.22%)	6 (1.34%)	13 (2.21%)	100 (9.40%)	28 (7.02%)
Skin	3 (0.64%)	0 (0.00%)	2 (0.66%)	9 (2.11%)	3 (0.16%)	0 (0.00%)	2 (0.45%)	1 (0.17%)	66 (6.20%)	29 (7.27%)
Other Non Sterile Culture	17 (3.63%)	8 (5.52%)	19 (6.27%)	25 (5.85%)	4 (0.21%)	3 (0.33%)	11 (2.46%)	9 (1.53%)	10 (0.94%)	2 (0.50%)
Other Sterile Culture	2 (0.43%)	1 (0.69%)	1 (0.33%)	3 (0.70%)	25 (1.31%)	13 (1.42%)	4 (0.89%)	14 (2.38%)	21 (1.97%)	11 (2.76%)

Characteristics	*Acinetobacter baumannii*		*Pseudomonas aeruginosa*		*Escherichia coli*		*Klebsiella pneumoniae*		*Staphylococcus aureus*	
	**CRAB**	**CSAB**	**CRPA**	**CSPA**	**3GCREC**	**3GCSEC**	**3GCRKP**	**3GCSKP**	**MRSA**	**MSSA**
**Number of multidrug-resistant isolates**										
MDR	462 (98.72%)	25 (17.24%)	257 (84.82%)	40 (9.37%)	1,670 (87.21%)	247 (27.05%)	416 (93.06%)	43 (7.30%)	981 (92.20%)	151 (37.84%)
Non-MDR	6 (1.28%)	120 (82.76%)	46 (15.18%)	387 (90.63%)	245 (12.79%)	666 (72.95%)	31 (6.94%)	546 (92.70%)	83 (7.80%)	248 (62.16%)
p-value [(a)]	<0.001		<0.001		<0.001		<0.001		<0.001	
**Polymicrobial, n (%)**										
2	166 (35.47%)	40 (27.59%)	132 (43.56%)	156 (36.53%)	122 (6.37%)	32 (3.50%)	147 (32.89%)	95 (16.13%)	115 (10.81%)	38 (9.52%)
3	61 (13.03%)	23 (15.86%)	51 (16.83%)	65 (15.22%)	33 (1.72%)	17 (1.86%)	74 (16.55%)	30 (5.09%)	58 (5.45%)	17 (4.26%)
4	19 (4.06%)	7 (4.83%)	18 (5.94%)	20 (4.68%)	11 (0.57%)	5 (0.55%)	14 (3.13%)	12 (2.04%)	17 (1.60%)	6 (1.50%)
5	0 (0.00%)	2 (1.38%)	1 (0.33%)	1 (0.23%)	2 (0.10%)	0 (0.00%)	0 (0.00%)	2 (0.34%)	1 (0.09%)	1 (0.25%)
p-value [(a)]	0.073		0.074		0.018		<0.001		0.67	
**Number of diagnoses, median (range)**	5 (2, 15)	2 (1, 5)	2 (2, 14)	2 (2, 7)	2 (2, 2)	2 (1, 2)	2 (2, 14)	2 (2, 5)	2 (1, 3)	2 (1, 2)
p-value [(b)]	<0.001		<0.001		0.27		<0.001		0.38	
**Antimicrobial Groups prescribed**										
J01CF (Beta-lactamase resistant penicillins)	11 (2.35%)	7 (4.83%)	9 (2.97%)	30 (7.03%)	25 (1.31%)	7 (0.77%)	10 (2.24%)	14 (2.38%)	407 (38.25%)	254 (63.66%)
p-value [(a)]	0.16		0.016		0.21		0.88		<0.001	
J01CR (Combinations of penicillins, incl. beta-lactamase inhibitors)	204 (43.59%)	60 (41.38%)	152 (50.17%)	162 (37.94%)	486 (25.38%)	212 (23.22%)	200 (44.74%)	155 (26.32%)	247 (23.21%)	93 (23.31%)
p-value [(a)]	0.64		0.001		0.21		<0.001		0.97	
J01DD (Third-generation cephalosporins)	209 (44.66%)	78 (53.79%)	156 (51.49%)	209 (48.95%)	823 (42.98%)	553 (60.57%)	218 (48.77%)	333 (56.54%)	257 (24.15%)	116 (29.07%)
p-value [(a)]	0.054		0.5		<0.001		0.013		0.055	
J01DH (Carbapenems)	422 (90.17%)	86 (59.31%)	280 (92.41%)	283 (66.28%)	1,300 (67.89%)	282 (30.89%)	362 (80.98%)	361 (61.29%)	283 (26.60%)	93 (23.31%)
p-value [(a)]	<0.001		<0.001		<0.001		<0.001		0.2	
J01EE (Combinations of sulfonamides and trimethoprim)	72 (15.38%)	15 (10.34%)	58 (19.14%)	53 (12.41%)	93 (4.86%)	22 (2.41%)	59 (13.20%)	21 (3.57%)	312 (29.32%)	37 (9.27%)
p-value [(a)]	0.13		0.013		0.002		<0.001		<0.001	
J01FA (Macrolides)	33 (7.05%)	8 (5.52%)	26 (8.58%)	19 (4.45%)	65 (3.39%)	19 (2.08%)	25 (5.59%)	11 (1.87%)	45 (4.23%)	16 (4.01%)
p-value [(a)]	0.52		0.022		0.054		0.001		0.85	
J01GB (Other aminoglycosides)	30 (6.41%)	20 (13.79%)	49 (16.17%)	45 (10.54%)	69 (3.60%)	15 (1.64%)	57 (12.75%)	20 (3.40%)	29 (2.73%)	23 (5.76%)
p-value [(a)]	0.005		0.025		0.004		<0.001		0.005	
J01MA (Fluoroquinolones)	175 (37.39%)	49 (33.79%)	156 (51.49%)	193 (45.20%)	435 (22.72%)	185 (20.26%)	160 (35.79%)	133 (22.58%)	272 (25.56%)	80 (20.05%)
p-value [(a)]	0.43		0.094		0.14		<0.001		0.028	
J01XA (Glycopeptide antibacterials)	213 (45.51%)	40 (27.59%)	144 (47.52%)	141 (33.02%)	142 (7.42%)	33 (3.61%)	135 (30.20%)	102 (17.32%)	643 (60.43%)	138 (34.59%)
p-value [(a)]	<0.001		<0.001		<0.001		<0.001		<0.001	
J01XB (Polymyxins)	319 (68.16%)	18 (12.41%)	176 (58.09%)	68 (15.93%)	62 (3.24%)	13 (1.42%)	206 (46.09%)	32 (5.43%)	52 (4.89%)	14 (3.51%)
p-value [(a)]	<0.001		<0.001		0.005		<0.001		0.26	

Note: J01CF (Beta-lactamase resistant penicillins), J01CR (Combinations of penicillins, incl. beta-lactamase inhibitors), J01DD (Third-generation cephalosporins), J01DH (Carbapenems), J01EE (Combinations of sulfonamides and trimethoprim, incl. derivatives), J01FA (Macrolides), J01GB (Other aminoglycosides), J01MA (Fluoroquinolones), J01XA (Glycopeptide antibacterials), J01XB (Polymyxins)<br>*p-value* [(a)] :Hypothesis testing results from the chi-squared or Fisher's exact test (for categorical data)<br>*p-value* [(b)]: Hypothesis testing results from the Mann-Whitney U test (for non-parametric data)
