# Supplementary figures and images for: Healthcare utilization associated with antimicrobial resistance at a tertiary hospital in Vietnam: A retrospective observational study from 2016 to 2021
Source: PLoS One. 2025 Aug 4;20(8):e0329539. doi: 10.1371/journal.pone.0329539 (PMC12321119; doi:10.1371/journal.pone.0329539)

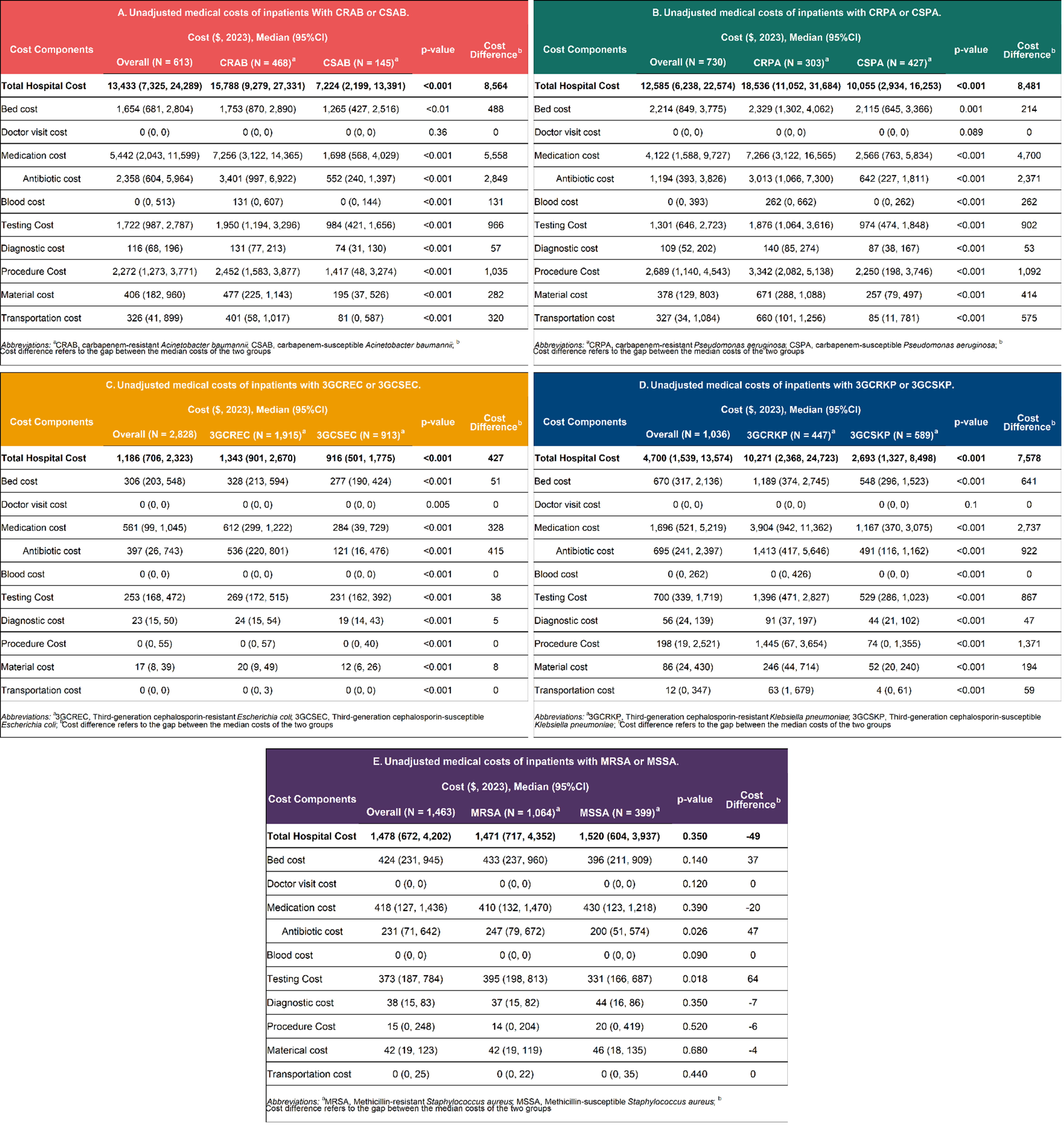

Supplement: S2 Fig — (TIF) [file pone.0329539.s002.tif]
