# Supplementary material for: Healthcare utilization associated with antimicrobial resistance at a tertiary hospital in Vietnam: A retrospective observational study from 2016 to 2021
Source: PLoS One. 2025 Aug 4;20(8):e0329539. doi: 10.1371/journal.pone.0329539 (PMC12321119; doi:10.1371/journal.pone.0329539)
Supplement: S3 Table — (PDF) [file pone.0329539.s003.pdf]

S3 Table. Results of multivariate generalized linear regression

Variables	Model 1<br>Marginal effect<br>(95%CI) ($,2023)	Model 2<br>Marginal effect<br>(95%CI) ($,2023)	Model 3<br>Marginal effect<br>(95%CI) ($,2023)	Model 4<br>Marginal effect<br>(95%CI) ($,2023)	Model 5<br>Marginal effect<br>(95%CI) ($,2023)
CRAB vs. CSAB	17,041***<br>(15,068 - 19,015)	13,362***<br>(11,822 - 14,902)	7,645***<br>(6,576 - 8,714)	5,576***<br>(4,561 - 6,592)	3,980***<br>(3,170 - 4,790)
CRPA vs. CSPA	20,368***<br>(16,998 - 23,737)	17,337***<br>(14,408 - 20,266)	10,628***<br>(8,056 - 13,200)	997*<br>(167 - 1,827)	1,000*<br>(225 - 1,775)
3GCREC vs. 3GCSEC	-490<br>(-1,513 - 533)	447<br>(-422 - 1,316)	461<br>(-279 - 1,200)	195<br>(-298 - 688)	444*<br>(6 - 894)
3GCRKP vs. 3GCSKP	15,474***<br>(13,090 - 17,857)	10,994***<br>(9,241 - 12,747)	5,877***<br>(4,843 - 6,912)	2,740***<br>(1,911 - 3,568)	1,942***<br>(1,212 - 2,672)
MRSA vs. MSSA	1,444**<br>(446 - 2,442)	2,295***<br>(1,208 - 3,381)	1,306***<br>(537 - 2,074)	-485<br>(-1,037 - 68)	-326*<br>(-806 - -154)
Period		27,672***<br>(13,284 - 42,060)	22,539***<br>(13,180 - 31,899)	4,403*<br>(910 - 7,896)	1163<br>(-1,101 - 3,427)
ASP		61***<br>(41 - 82)	11<br>(-7 - 30)	8<br>(-3 - 20)	4<br>(-7 - 15)
COVID		-2,811***<br>(-4,283 - -1,339)	-379<br>(-1,382 - 624)	-508<br>(-1,258 - 241)	154<br>(-421 - 729)
**Gender**					
Female vs. Male		-3,544***<br>(-4,352 - -2,736)	-2,583***<br>(-3,202 - -1,965)	-1,753***<br>(-2,192 - -1,315)	-1,534***<br>(-1,917 - -1,151)
**Age**		38***<br>(20 - 56)	14<br>(-2 - 31)	31***<br>(19 - 43)	10<br>(-1 - 22)
**Admission to ICU**					
ICU vs. No ICU			12,145***<br>(11,295 - 12,995)		5,301***<br>(4,719 - 5,884)
**Health insurance benefit levels**					
80% vs. No Insurance level			1,879***<br>(1,027 - 2,731)		741**<br>(259 - 1,224)
95% vs. No Insurance level			4,987***<br>(2,283 - 7,691)		3,192***<br>(1,858 - 4,526)
100% vs. No Insurance level			2,402***<br>(1,326 - 3,477)		1,181***<br>(570 - 1,792)
**Type of infection**					
COVID-19 vs. bloodstream infection (BSI)				8,668***<br>(4,855 - 12,482)	11,436***<br>(7,660 - 15,213)

Variables	Model 1<br>Marginal effect<br>(95%CI) ($,2023)	Model 2<br>Marginal effect<br>(95%CI) ($,2023)	Model 3<br>Marginal effect<br>(95%CI) ($,2023)	Model 4<br>Marginal effect<br>(95%CI) ($,2023)	Model 5<br>Marginal effect<br>(95%CI) ($,2023)
HIV vs. BSI				-2,336***<br>(-2,895 - -1,776)	-1,651***<br>(-2,193 - -1,109)
Intra-abdominal infection vs. BSI				-1,129***<br>(-1,753 - -505)	-847**<br>(-1,442 - -252)
Lower respiratory tract infection (LRTI) vs. BSI				2,781***<br>(1,313 - 4,248)	1,864*<br>(442 - 3,285)
Meningitis vs. BSI				3,228***<br>(1,447 - 5,009)	1,640*<br>(78 - 3,203)
Skin Infection vs. BSI				-4,041***<br>(-4,586 - -3,495)	-3,392***<br>(-3,878 - -2,907)
Tetanus vs. BSI				2,677***<br>(2,070 - 3,285)	753*<br>(55 - 1,452)
Urinary tract infection vs. BSI				-3,751***<br>(-4,242 - -3,261)	-3,584***<br>(-4,039 - -3,130)
Other diseases vs. BSI				586<br>(-126 - 1,297)	-434<br>(-1,062 - 194)
**Charlson Comorbidity Index (CCI)**					
CCI 1 vs. CCI 0				-152<br>(-1,671 - 1,367)	-385<br>(-1,577 - 807)
CCI 2 vs. CCI 0				2,390***<br>(1,701 - 3,078)	2,196***<br>(1,588 - 2,804)
CCI >=3 vs. CCI 0				599<br>(-247 - 1,445)	843*<br>(44 - 1,642)
**Treatment outcomes**					
Unchanged vs. Improved				3,171***<br>(2,364 - 3,977)	2,034***<br>(1,324 - 2,743)
Worsen vs. Improved				13,607***<br>(11,877 - 15,337)	8,187***<br>(6,905 - 9,468)
Deceased vs. Improved				9,104***<br>(6,625 - 11,582)	7,926***<br>(5,993 - 9,859)
**Length of stay**				571***<br>(518 - 625)	508***<br>(459 - 558)
AIC	19.104	18.958	18.568	18.138	18.093
BIC	-47456.36	-48364.70	-50829.84	-53529.94	-53771.01
Pseudo R-squared	0.031	0.038	0.060	0.097	0.107

Note: Model 1: unadjusted generalized linear model. Model 2, 3, 4, 5: linear splines with one knot in June 2021 and generalized linear regression models. Model 2: adjusted for age and sex; Model 3: adjusted for sociodemographic variables (gender, age, health insurance benefit levels, admission to ICU). Model 4: adjusted for age, sex, and clinical variables (type of infection, Charlson Comorbidity Index , LOS, treatment outcomes). Model 5: a fully adjusted model for all sociodemographic and clinical variables and the interaction between *A.baumannii* and *K.pneumoniae*. * $p < 0.05$, ** $p < 0.01$, *** $p < 0.001$. CI – Confidence interval
