# Supplementary material for: Healthcare utilization associated with antimicrobial resistance at a tertiary hospital in Vietnam: A retrospective observational study from 2016 to 2021
Source: PLoS One. 2025 Aug 4;20(8):e0329539. doi: 10.1371/journal.pone.0329539 (PMC12321119; doi:10.1371/journal.pone.0329539)
Supplement: S4 Table — (PDF) [file pone.0329539.s004.pdf]

*S4 Table. Results of multivariate negative binomial regression*

| Variables | Model 1<br>Marginal effect (95%CI) (days) | Model 2<br>Marginal effect (95%CI) (days) | Model 3<br>Marginal effect (95%CI) (days) | Model 4<br>Marginal effect (95%CI) (days) | Model 5<br>Marginal effect (95%CI) (days) |
|---|---|---|---|---|---|
| *CRAB vs. CSAB* | 12.31***<br>(10.99 - 13.63) | 12.43***<br>(10.99 - 13.87) | 7.00***<br>(5.80 - 8.19) | 8.65***<br>(7.47 - 9.82) | 7.07***<br>(5.90 - 8.23) |
| *CRPA vs. CSPA* | 22.50***<br>(20.74 - 24.25) | 20.92***<br>(19.14 - 22.69) | 14.06***<br>(12.68 - 15.44) | 14.00***<br>(12.43 - 15.57) | 12.54***<br>(11.12 - 13.97) |
| *3GCREC vs. 3GCSEC* | 0.24<br>(-0.63 - 1.10) | 0.94*<br>(0.04 - 1.84) | 0.90*<br>(0.13 - 1.68) | 1.63***<br>(0.86 - 2.39) | 1.40***<br>(0.69 - 2.10) |
| *3GCRKP vs. 3GCSKP* | 10.12***<br>(8.70 - 11.54) | 10.09***<br>(8.62 - 11.56) | 6.51***<br>(5.28 - 7.74) | 7.97***<br>(6.71 - 9.23) | 6.80***<br>(5.57 - 8.04) |
| *MRSA vs. MSSA* | 5.23***<br>(4.14 - 6.32) | 5.04***<br>(3.94 - 6.14) | 4.77***<br>(3.82 - 5.72) | 5.06***<br>(4.06 - 6.06) | 4.96***<br>(4.02 - 5.91) |
| *Period* | | 10.49***<br>(4.91 - 16.07) | 9.87***<br>(5.05 - 14.68) | 3.48<br>(-2.05 - 9.02) | 0.22<br>(-5.51 - 5.95) |
| *ASP* | | 0.03**<br>(0.01 - 0.05) | -0.01<br>(-0.03 - 0.01) | 0<br>(-0.02 - 0.02) | -0.02*<br>(-0.04 - -0.00) |
| *COVID* | | -3.47***<br>(-4.76 - -2.18) | -1.55**<br>(-2.66 - -0.43) | -2.75***<br>(-3.75 - -1.75) | -1.48**<br>(-2.42 - -0.54) |
| **Gender** | | | | | |
| *Female vs. Male* | | -4.44***<br>(-5.18 - -3.69) | -3.24***<br>(-3.89 - -2.58) | -2.02***<br>(-2.66 - -1.37) | -1.87***<br>(-2.48 - -1.26) |
| **Age** | | 0.04***<br>(0.02 - 0.06) | 0<br>(-0.01 - 0.02) | 0.02*<br>(0.00 - 0.04) | 0<br>(-0.01 - 0.02) |
| **Admission to ICU** | | | | | |
| *ICU vs. No ICU* | | | 13.01***<br>(11.87 - 14.15) | | 8.02***<br>(6.78 - 9.26) |
| **Health insurance benefit levels** | | | | | |
| *80% vs. No Insurance level* | | | 2.29***<br>(1.51 - 3.07) | | 2.20***<br>(1.48 - 2.91) |
| *95% vs. No Insurance level* | | | 4.43***<br>(2.28 - 6.59) | | 4.22***<br>(2.29 - 6.15) |
| *100% vs. No Insurance level* | | | 2.98***<br>(1.96 - 4.00) | | 2.54***<br>(1.61 - 3.46) |
| **Type of infection** | | | | | |
| *COVID-19 vs. bloodstream infection (BSI)* | | | | 10.00***<br>(13.06 - 26.95) | 11.76***<br>(12.69 - 30.83) |
| *HIV vs. BSI* | | | | 1.31*<br>(0.09 - 2.53) | 2.15***<br>(0.88 - 3.41) |
| *Intra-abdominal infection vs. BSI* | | | | 0.36<br>(-0.70 - 1.42) | 0.48<br>(-0.57 - 1.54) |
| *Lower respiratory tract infection (LRTI) vs. BSI* | | | | 8.42***<br>(6.16 - 10.69) | 5.97***<br>(3.90 - 8.04) |
| *Meningitis vs.BSI* | | | | 19.93***<br>(17.08 - 22.78) | 14.82***<br>(12.29 - 17.35) |
| *Skin Infection vs. BSI* | | | | -3.71***<br>(-4.56 - -2.86) | -3.08***<br>(-3.92 - -2.23) |
| *Tetanus vs. BSI* | | | | 19.99***<br>(18.76 - 21.23) | 16.80***<br>(15.33 - 18.26) |

| Variables | Model 1 Marginal effect (95%CI) (days) | Model 2 Marginal effect (95%CI) (days) | Model 3 Marginal effect (95%CI) (days) | Model 4 Marginal effect (95%CI) (days) | Model 5 Marginal effect (95%CI) (days) |
|---|---|---|---|---|---|
| *Urinary tract infection vs. BSI* | | | | -3.92*** (-4.52 - -3.31) | -3.83*** (-4.42 - -3.23) |
| *Other diseases vs. BSI* | | | | 7.83*** (6.51 - 9.16) | 5.71*** (4.53 - 6.89) |
| **CCI** | | | | | |
| *CCI 1 vs. CCI 0* | | | | 0.63 (-0.86 - 2.12) | 0.07 (-1.40 - 1.54) |
| *CCI 2 vs. CCI 0* | | | | 1.91*** (0.95 - 2.87) | 1.58*** (0.67 - 2.49) |
| *CCI >=3 vs. CCI 0* | | | | 2.52** (0.93 - 4.10) | 2.59*** (1.09 - 4.09) |
| **Treatment outcomes** | | | | | |
| *Unchanged vs. Improved* | | | | -2.87*** (-4.00 - -1.74) | -4.35*** (-5.42 - -3.28) |
| *Worsen vs. Improved* | | | | 2.74** (1.08 - 4.39) | -1.92** (-3.36 - -0.49) |
| *Deceased vs. Improved* | | | | -8.81*** (-10.23 - -7.39) | -9.24*** (-10.53 - -7.96) |
| *Pseudo R-squared* | 0.0423 | 0.0470 | 0.0708 | 0.901 | 0.988 |

Note: Model 1: unadjusted negative binomial regression model. Model 2, 3, 4, 5: linear splines with one knot in June 2021 and negative binomial regression models. Model 2: adjusted for age and sex, Model 3: adjusted for sociodemographic variables (gender, age, health insurance benefit levels, admission to ICU). Model 4: adjusted for age, sex, and clinical variables (type of infection, CCI, treatment outcomes). Model 5: a fully adjusted model for all sociodemographic and clinical variables and the interaction between *A.baumannii* and *K.pneumoniae*. * p < 0.05, ** p < 0.01, *** p < 0.001. CI – Confidence interval
